# Phase Matching via Plasmonic Modal Dispersion for Third Harmonic Generation

**DOI:** 10.1002/advs.202201180

**Published:** 2022-06-05

**Authors:** Zhe Wang, Zhe Wang, Vijith Kalathingal, Yi Wei Ho, Thanh Xuan Hoang, Hong‐Son Chu, Yongxin Guo, José C. Viana‐Gomes, Goki Eda, Christian A. Nijhuis

**Affiliations:** ^1^ Department of Chemistry National University of Singapore 3 Science Drive 3 Singapore 117543 Singapore; ^2^ Department of Physics National University of Singapore 2 Science Drive 3 Singapore 117542 Singapore; ^3^ Department of Electrical and Computer Engineering National University of Singapore 4 Engineering Drive 3 Singapore 117583 Singapore; ^4^ Department of Electronics and Photonics Institute of High Performance Computing A*STAR (Agency for Science, Technology and Research) 1 Fusionopolis Way, #16‐16 Connexis Singapore 138632 Singapore; ^5^ Department of Physics of University of Minho, and Physics Center of Minho and Porto Universities (CF‐UM‐UP), Campus of Gualtar Braga 4710‐057 Portugal; ^6^ Centre for Advanced 2D Materials and Graphene Research Centre National University of Singapore 6 Science Drive 2 Singapore 117546 Singapore; ^7^ Hybrid Materials for Opto‐Electronics Group Department of Molecules and Materials MESA+ Institute for Nanotechnology and Center for Brain‐Inspired Nano Systems Faculty of Science and Technology University of Twente AE Enschede 7500 The Netherlands

**Keywords:** modal phase matching, nonlinear polymers, plasmonic modes, plasmonic waveguides, third‐harmonic generation, two‐wire transmission lines

## Abstract

The overall effectiveness of nonlinear optical processes along extended nonlinear media highly depends on the fulfillment of the phase‐matching condition for pump and generated fields. This is traditionally accomplished by exploiting the birefringence of nonlinear crystals requiring long interaction lengths (cm‐scale). For nonbirefringent media and integrated photonic devices, modal phase matching can compensate the index mismatch. Here, the various interacting waves propagate in transverse modes with appropriate phase velocities, but they suffer from a low refractive index contrast and cm‐scale interaction lengths. This work harnesses modal phase matching for third‐harmonic generation (THG) in plasmonic waveguides using an organic polymer (poly[3‐hexylthiophene‐2,5‐diyl]) as the nonlinear medium. One demonstrates experimentally an effective interaction area as small as ≈ 0.11 µm^2^ and the phase‐matched modal dispersion results in THG efficiency as high as ≈ 10^–3^ W^‐2^ within an effective length scale of ≈ 4.3 µm. THG also shows a strong correlation with the polarization of the incident laser beam, corresponding to the excitation of the antisymmetric plasmonic modes, corroborating that plasmonic modal phase matching is achieved. This large reduction in device area of orders of magnitude is interesting for various applications where space is critical (e.g., device integration or on‐chip applications).

## Graphical abstract

Plasmonic modal dispersion is exploited to fulfill the required phase matching condition for third‐harmonic generation in short (≈ 4.3 µm long) waveguides. In a two‐wire transmission line configuration, antisymmetric plasmonic gap modes mediate the nonlinear parametric process and the incident laser polarization dictates the third harmonic power, with excellent agreement between experiment and theory.

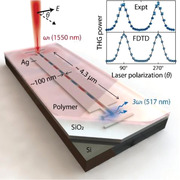

## 1 Introduction

Nonlinear optical processes have garnered a renewed interest in a multitude of applications ranging from all‐optical signal processing,^[^ ^1^, ^2^ ^]^ sensing,^[^ ^3^ ^]^ multiphoton microscopy,^[^ ^4^ ^]^ photocatalysis,^[^ ^5^, ^6^ ^]^ to quantum optics.^[^ ^7^ ^]^ Recently, optical frequency conversion, third‐harmonic generation (THG) in particular, has gained extensive research significance in spectroscopy^[^ ^8^ ^]^ and biological and medical applications,^[^ ^9^, ^10^, ^11^ ^]^ along with the demand for telecom to visible wavelength conversion capabilities.^[^ ^12^, ^13^ ^]^ Compared to conventional optical methods, an integrated micrometer‐scale device for nonlinear conversion, however, demands a major improvement in the design due to the inevitable long interaction length requirement between the pump and generated fields. In harmonic generation processes, these fields are typically separated by hundreds of nanometers, and an in‐phase build‐up of the field amplitudes is usually achieved by birefringent nonlinear crystals at a centimeter scale.^[^ ^14^ ^]^ Therefore, the potential for high‐density integration for on‐chip nanoscale optical conversions is compromised in these methods. Here we demonstrate phase‐matched modal dispersion in plasmonic waveguides as an alternative solution to improve the coherence length *l*_coh_ = *π*/Δ*k*, by minimizing the momentum mismatch Δ*k* between the interacting fields, and consequently the interaction length, to attain good nonlinear conversion efficiencies for THG. We show that the material's dispersion is compensated by a judiciously chosen geometry to match the phase velocity of the pump and generated fields, allowing us to demonstrate an effective interaction area as small as ≈ 0.11 µm^2^ and THG efficiency as high as ≈ 10^–3^ W^‐2^ which is comparable to other devices based on nonlinear optical polymers inside short waveguides (≈ 4.3 µm long). This is an order‐of‐magnitude reduction in device footprint compared to photonic devices^[^ ^15^, ^16^ ^]^ and is potentially useful for applications with stringent space requirements and device integration.

Plasmonics offers a versatile route to downscale the nonlinear components for the realization of active photonic circuitry with device designs in subwavelength dimensions.^[^ ^17^, ^18^ ^]^ The field confinements and the associated large field amplitudes allow for coherent enhancement of inherently weak nonlinear light–matter interaction in nanoscale systems.^[^ ^19^, ^20^, ^21^ ^]^ While plasmonic metals, in particular gold, exhibit very high third‐order susceptibilities,^[^ ^22^ ^]^ their nonlinear response is generally limited by the intrinsic electron damping and electromagnetic screening, especially in the visible and near‐infrared energy ranges.^[^ ^23^ ^]^ Plasmon‐mediated nonlinear conversion has been achieved in the past in 2D material systems^[^ ^12^ ^]^ and colloidal nanorods,^[^ ^24^, ^25^, ^26^, ^27^ ^]^ but at the cost of active interaction volume or limited flexibility in the device design. In comparison, nonlinear optical polymers (NOPs) offer solution‐based processing and large nonlinear susceptibilities via chemical modification or changing the structure of the polymer films. Their fabrication protocols are further suited for integrated nonlinear devices,^[^ ^28^, ^29^, ^30^, ^31^ ^]^ such as silicon photonics structures for applications in nonlinear optics, plasmonic phase modulation,^[^ ^32^ ^]^ and all‐optical signal processing.^[^ ^33^, ^34^ ^]^ Recently, four‐wave mixing was demonstrated using NOP as the nonlinear medium in a plasmonic slot waveguide.^[^ ^35^ ^]^ In this case, however, phase matching was not a concern as *l*_coh_, for small Δ*k*, was much larger than the waveguide length.

**Figure 1:**
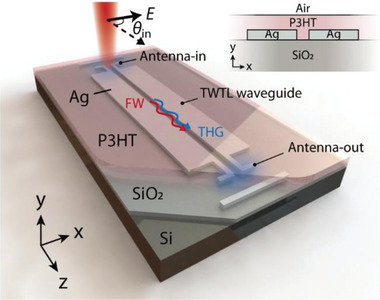
Schematic illustration of the device consisting of a TWTL plasmonic waveguide and two plasmonic antennas. The incident laser polarization angle *θ*_in_ is defined with respect to the orientation of the waveguide. The inset shows the cross‐section of the waveguide with P3HT layer as the nonlinear medium.

In this study, we demonstrate THG in plasmonic waveguides in a two‐wire transmission line (TWTL) configuration,^[^ ^36^ ^]^ where two Ag slabs are placed in nanoscale proximity with a NOP cladding as the active medium, as illustrated in **Figure** **1**. This type of platform supports symmetric (S) and antisymmetric (AS) plasmonic modes^[^ ^37^ ^]^ and is in favor of modal phase matching (MPM),^[^ ^14^, ^38^ ^]^ with operation wavelengths extending from the optical to the near‐infrared spectral range. We used poly(3‐hexylthiophene‐2,5‐diyl) (P3HT) as NOP for the conversion of the telecommunication wavelength (1550 nm) to its third harmonic (TH) (517 nm). This NOP has a large third‐order nonlinear susceptibility *χ* ^(3)^ ≈ 10^−19^ m^2^V^−2^.^[^ ^39^ ^]^ We fabricated a series of devices with different waveguide lengths (*L*) and gap widths (*W*), carefully designed to achieve MPM with a good overlap between the two AS eigenmodes: one at the fundamental wavelength (FW) 1550 nm and the other at 517 nm. By focusing a 1550 nm wavelength pump laser on one of the antennas (Antenna‐in, Figure 1), we found a significant THG output at 517 nm at the other end of the waveguide (Antenna‐out, Figure 1). The polarization angle (*θ*_in_) of the incident laser beam shows a strong correlation with the THG signal intensity, an observation consistent with the excitation of the AS plasmonic modes in nonlinear energy conversion and further verified with the nonlinear finite difference time domain (FDTD) simulations. The results presented here pave the way to a nanophotonic device element for telecom‐to‐visible nonlinear optical conversion, but are also useful in other areas where space is limited.

## 2 Results and Discussion

### 2.1 Waveguide Design and Modal Phase Matching

**Figure 2:**
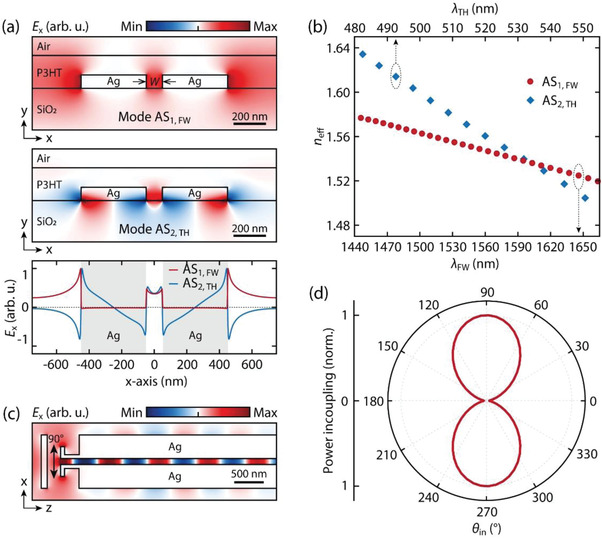
Eigenmode analysis and the mode excitation for the waveguide segment. a) Modal field profile (*E*_x_) of the eigenmodes supported by the waveguide for AS_1, FW_ (top) and AS_2, TH_ (mid). The bottom panel shows the *E*_x_ obtained from the top and middle panels at *y* = 0 (middle of the waveguide) plotted as a function *x*. b) Real part of the effective refractive index (*n*_eff_) for the eigenmodes AS_1, FW_ (bottom *x*‐axis) and AS_2, TH_ (top *x*‐axis) as a function of wavelength. c) Simulated electric field (*E*_x_) in the waveguide excited by a Gaussian beam with *θ*_in_ = 90°. d) Polar plot for the simulated in‐coupled power (normalized to the maxima) for *λ* = 1550 nm as a function of the laser polarization angle *θ*_in_, which follows a sin^2^ *θ*_in_ with a maximal mode power coupled at *θ*_in_ = 90° and 270°.

The plasmonic waveguides investigated here consist of two Ag slabs in TWTL configuration (Figure 1 inset) on SiO_2_ substrate (285 nm thick on a Si wafer), with the width and height of the slabs fixed as 400 and 80 nm respectively. The gap width *W* is varied from 100 to 200 nm (see Section S1, Supporting Information). For the slabs, Ag is preferred as the plasmonic material, which has a lower interband absorption loss around the THG wavelength (517 nm in this work) than Au.^[^ ^40^ ^]^ The NOP (here P3HT) was spin‐coated over the structure to a thickness of around 200 nm, filling the gap between the two slabs (see Figure S1, Supporting Information for AFM images). In the TWTL configuration, the electric field inside the slot is dominated by the AS modes, whereas the S modes (not being investigated in this work) are delocalized outside the Ag slabs.^[^ ^37^ ^]^ Therefore, we consider only the first‐order AS mode for FW and the second‐order AS mode for THG, which are depicted in **Figure** **2**a as AS_1, FW_ (top panel) and AS_2, TH_ (middle panel), respectively. The eigenmode analysis and mode dispersion calculations were performed using MODE‐solutions of the commercial FDTD package Lumerical.^[^ ^41^ ^]^ The mode orders are designated according to their charge distribution in the Ag slabs (see Figure S3, Supporting Information for the charge distribution plot). We note that the field intensity inside the Ag slabs is suppressed by the electromagnetic screening, leading to a negligible contribution to the THG signal. For the THG process, the effective interaction area between the AS_1, FW_ mode and the P3HT medium is crucial and is quantified as the ratio of the integrated electromagnetic power in the transverse plane (*xy*‐plane) to the effective intensity in the nonlinear medium (P3HT):^[^ ^42^ ^]^

(1) Aeff=Z02nP3HT2∫∫x,yReEx,y×H∗x,y·z^dxdy2∫∫P3HTEx,y4dxdy

where *Z*_0_ is the free space impedance, $\hat{z}$ is the unit vector orthogonal to the *xy*‐plane and *n*_P3HT_ is the linear refractive index of the NOP. Plasmonic mode confinement warrants *A*_eff_ as small as ≈ 0.11 µm^2^ for *W* = 100 nm, as estimated from the FDTD calculations (*A*_eff_ for all values of *W* are listed in Table S1, Supporting Information). Good overlap between the two AS modes inside the waveguide gap (Figure 2a, bottom panel) enables efficient coupling to the AS_2, TH_ mode of THG photons radiated by the nonlinear polarization created in the P3HT by the FW field propagating in the AS_1, FW_ mode.

Along with this mode overlap, the efficiency of the THG process depends critically on phase matching between FW and TH eigenmodes and we turn now to the discussion of the MPM mechanism. Important results obtained from the FDTD calculation are the complex effective indices $n_{\mathrm{eff}}^{\alpha }\left(\lambda \right)+i\kappa_{\mathrm{eff}}^{\alpha }\left(\lambda \right)$ for the two AS modes with *α* standing for FW and TH. Figure 2b shows $n_{\mathrm{eff}}^{\alpha }\left(\lambda \right)$ as a function of wavelength for the AS_1, FW_ mode (bottom *x*‐axis) and the AS_2, TH_ mode (top *x*‐axis) for the case of *W* = 100 nm. The phase mismatch between the FW and the TH eigenmodes is defined as Δ*k* = 3*k* ^FW^ − *k* ^TH^, where $k^{\alpha }=2\pi n_{\mathrm{eff}}^{\alpha }\left(\lambda_{\alpha }\right)/\lambda_{\alpha }$ is the wavenumber of the *α*‐eigenmode, with *λ*_*α*_ the corresponding wavelength. As for THG *λ*_FW_ = 3*λ*_TH_, then $\Delta k=\frac{2\pi }{\lambda_{\mathrm{TH}}}\left[n_{\mathrm{eff}}^{\mathrm{FW}}\left(\lambda_{\mathrm{FW}}\right)-n_{\mathrm{eff}}^{\mathrm{TH}}\left(\lambda_{\mathrm{TH}}\right)\right]$, showing that the MPM condition Δ*kL* ≪ 1 is attained when the two modes have the same effective index, where *L* represents the length of the waveguide. Figure 2b shows that the MPM is achieved for the AS_1, FW_ and the AS_2, TH_ at the crossing point of the dispersion curves at around 1600 nm. In this study, the AS_1, FW_ and AS_2, TH_ modes wavelengths are 1550 and 517 nm and the difference between the effective indices is about ≈ 0.015, which corresponds to *l*_coh_ ≈ 17 µm, for *W* = 100 nm.

To couple the pump beam into the AS modes from free space and to couple out the THG signal from the waveguide end, we use dipole‐like plasmonic antennas (Antenna‐in and Antenna‐out respectively, Figure 1) oriented perpendicularly to the waveguide. Such an antenna structure offers two advantages: i) it has a polarization angle *θ*_in_ (defined with respect to the waveguide orientation in Figure 1) dependent mode selectivity for the excitation of AS or S modes,^[^ ^37^ ^]^ and ii) it allows for nearly vertical outcoupling for the guided THG modes, which can be captured by an objective lens in the reflection mode (see Figure S4d, Supporting Information). A linearly polarized pump beam is introduced through Antenna‐in at normal incidence. For *θ*_in_ = 90°, the coupling to the AS modes is maximal and a propagating plasmonic field is established inside the waveguide as shown in Figure 2c, where *E_x_* is the *x* component of the complex electric field amplitude evaluated at 1550 nm. The polar plot in Figure 2d shows the electromagnetic power coupled into the waveguide (normalized to the maximum at *θ*_in_ = 90°) for *λ* = 1550 nm calculated as a function of *θ*_in_; it follows a sin^2^ *θ*_in_ dependence with two maxima at 90^○^ and 270^○^, which corresponds to the direction colinear with the antenna dipole.

### 2.2 Nonlinear Optical Measurements

**Figure 3:**
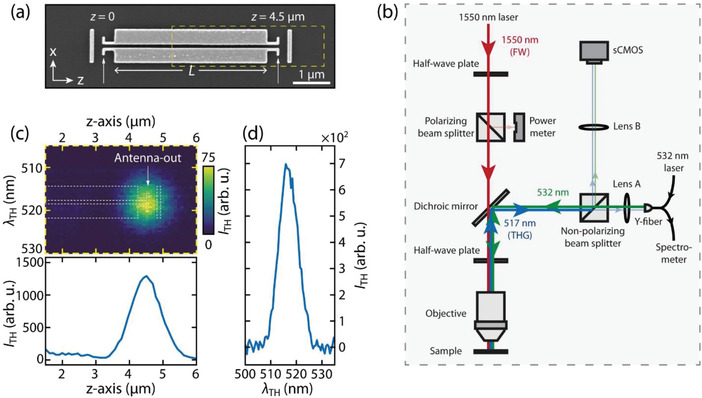
Spectroscopic THG measurements. a) SEM image for the representative device with a waveguide length *L* = 4.0 µm and a gap width *W* = 100 nm. b) Schematic illustration of the optical setup for recording the THG spectra and the images. The nonconfocal THG spectroscopy is realized by moving the fiber coupler on the image plane using a piezo‐controlled Y‐fiber detection system. The injection of a 532 nm CW laser in one of the ends of the Y‐fiber allows precise determination of the location where the signal is acquired through direct visualization of the green spot in the sCMOS camera. Part of the THG signal is routed to a spectrometer by the other Y‐end of the fiber. c) Intensity map of the line‐trace spectra (top). Integrated THG signal as a function of detection position along the waveguide (bottom). d) THG spectra from the Antenna‐out location, corresponding to the detection position at *z* = 4.5 µm.

**Figure** **3**a shows the scanning electron microscope (SEM) image of a representative device with *L* = 4 µm and *W* = 100 nm, and the Antenna‐in is located at *z* = 0. The devices were fabricated on a SiO_2_‐Si substrate with standard electron‐beam lithography and evaporation techniques and the P3HT polymer film was spin‐coated on the waveguide (See Experimental Section for details). The schematic illustration of the experimental setup for the nonlinear optical measurements is shown in Figure 3b. To obtain the spatially resolved nonconfocal mapping for the THG signals, we focus the laser beam (1550 nm, FW) on the Antenna‐in through an objective lens while scanning the THG signal collection spot along the waveguide (i.e., *z*‐axis). The acquisition of signal collection location is realized using a Y‐fiber equipped with a laser diode as an indicator. The input 1550 nm pump laser intensity (*P*_in_) is monitored and controlled by the combination of a half‐wave plate, a polarizing beam splitter, and a power meter. The angle of excitation polarization (*θ*_in_) is controlled by another half‐wave plate before the objective (see Experimental Section for details).

The THG intensity (*I*_TH_) for *θ*_in_ = 90°, collected along the length of the waveguide (corresponding to the dashed‐line box shown in Figure 3a) as a function of the wavelength is plotted in Figure 3c (top). In the bottom panel of Figure 3c the spectrally integrated *I*_TH_ along the waveguide length is plotted to show a maximum intensity at the Antenna‐out location. Figure 3d shows the wavelength resolved spectra of the *I*_TH,_ collected at *z* = 4.5 µm (Antenna‐out) and centered at 517 nm. Under the laser excitation of 1550 nm with *θ*_in_ = 90°, the AS_1, FW_ mode of the waveguide is excited and the third‐order P3HT isotropic susceptibility $\chi_{\mathrm{TH}}^{\left(3\right)}\equiv \chi^{\left(3\right)}\left(3\omega =\omega +\omega +\omega \right)$ induces a nonlinear polarization^[^ ^14^ ^]^ $P_{x}^{\left(3\right)}\left(3\omega \right)=\varepsilon_{0}\chi_{\mathrm{TH}}^{\left(3\right)}{\left[E_{x}\left(\omega \right)\right]}^{3}$ in the waveguide transverse direction (where *ε*_0_ is the permittivity of free space and *E_x_* (*ω*) is the *x* component of the complex amplitude of the electric field of the AS_1, FW_ mode at frequency *ω*), which generates the AS_2, TH_ mode, phase‐matched with the AS_1, FW_ mode, thus resulting in the *I*_TH_ at the Antenna‐out centered at the wavelength 517 nm.

**Figure 4:**
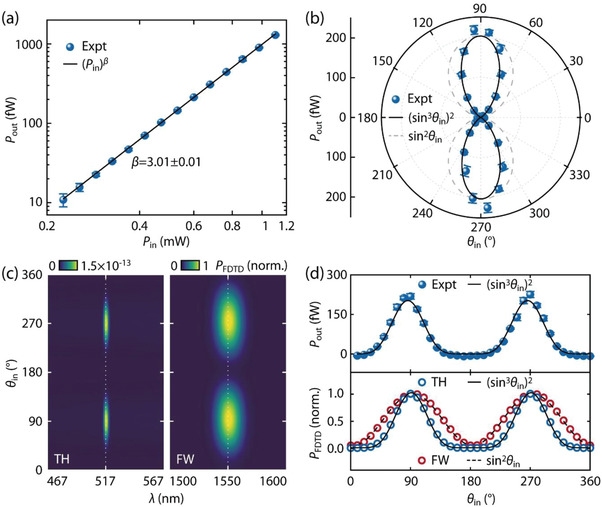
a) Output THG power (*P*_out_) from the Antenna‐out as a function of average *P*_in_. The black solid line is a power‐law fit $P_{\mathrm{in}}^{\beta }$. b) *P*_out_ as a function of *θ*_in_ for *P*_in_ = 0.6 mW. The black solid line is (sin^3^ *θ*_in_)^2^ function fitted to the experimental data and the gray dashed curve shows sin^2^ *θ*_in_ function. The error bars show the standard deviation of five repeated measurements on the same device. c) Nonlinear FDTD simulation results for the power (*P*_FDTD_) at the Antenna‐out location, evaluated as a function of the *θ*_in_ and *λ*. *P*_FDTD_ is normalized to the maximum value at 1550 nm. d) Comparison of the output power from the experiment (*P*_out_, top) and the nonlinear FDTD simulations (*P*_FDTD_, bottom) as a function of *θ*_in_. Black solid and dashed lines are the fits to (sin^3^ *θ*_in_)^2^ and sin^2^ *θ*_in_ functions respectively.

The third‐order character of the nonlinear process is further confirmed by measuring the pump intensity (*P*_in_) dependence of the integrated output THG power (*P*_out_) as shown in **Figure** **4**a: the power‐law fit $P_{\mathrm{in}}^{\beta }$ to the experimental data yields *β* ≈ 3 as expected.^[^ ^14^ ^]^ For *P*_in_ in the range of 0.22–1.1 mW, we observe *P*_out_ ranging between 11 and 1300 fW from the Antenna‐out location. This gives an average THG power conversion efficiency *η* (*P*_out_/(*P*_in_)^3^) ≈ 10^–3^ W^‐2^ (see Experimental Section for details). We restrict the unit of *η* to W^–2^ instead of W^–2^ m^–1^ to avoid inconsistent normalization of *η* with *L*, as the plasmonic losses make the FW power along the waveguide an attenuating function of *L*. Figure 4b shows the angular dependence of *P*_out_ as a function of *θ*_in_. The minimum in the polar plot corresponds to *P*_out_ for polarization along the *z*‐axis ( *θ*_in_ = 0° or 180°), where only the S modes of the plasmonic waveguide are excited.^[^ ^37^ ^]^ For *θ*_in_ = 90° or 270°, the AS_1, FW_ mode (Figure 2a, top) is efficiently excited, with major field intensity localized within the waveguide gap, leading to the conversion to the AS_2, TH_ mode (Figure 2a, middle) from the nonlinear interaction with the P3HT medium. Therefore, we ascribe this strong dependence of the laser polarization as a direct evidence of the plasmonic MPM in our waveguide. The third order character of *P*_out_ with the variation in *θ*_in_ is further evidenced from the ${\left(\mathrm{si}n^{3}\theta_{\mathrm{in}}\right)}^{2}\propto |E_{x}^{3}{|}^{2}$ fitting (solid line), in contrast with the sin^2^ *θ*_in_∝|*E*_x_|^2^ dependence (dashed line) which would be expected for a linear response. Here *E*_x_ represents the *x*‐component of the incident laser field.

The plasmonic MPM efficacy was also checked by nonlinear FDTD simulations. A Gaussian beam was used as the input source for the FW excitation (1550 nm) with a pulse duration of 100 fs and a field peak amplitude of | ***E*** | ≈ 10^7^ V m^‐1^, such that the third‐order term (*χ* ^(3)^| ***E*** |^2^ ≈ 10^–5^) is a perturbation to the linear response (see Experimental and Simulation Section for more details). Figure 4c shows the power outcoupled (*P*_FDTD_) from Antenna‐out as a function of the wavelength (*x*‐axis) and *θ*_in_ (*y*‐axis) for the waveguide with *L* ≈ 4 µm and *W* = 100 nm. *P*_FDTD_ is normalized to the maximum value at 1550 nm. The maxima observed at *θ*_in_ = 90° and 270° for wavelengths 1550 and 517 nm respectively correspond to the outcoupling of the FW and TH. To compare with the experimental data, *P*_FDTD_ values along the dotted lines in Figure 4c (at 1550 and 517 nm and normalized to unity individually) are plotted as a function of *θ*_in_ in the bottom panel of Figure 4d. The third order nature of the THG process due to the antisymmetric mode AS_1, FW_ is evident from the (sin^3^ *θ*_in_)^2^ fitting (solid line). The peak positions (at *θ*_in_ = 90° and 270°) correspond to the excitation of the AS modes in the plasmonic waveguide, consistent with the experimental result (blue spheres, top panel). The variation of *P*_FDTD_ at FW (red circles, bottom panel) follows the projection of the laser polarization on the antenna direction in a sin^2^ *θ*_in_ form (dashed line, bottom panel), consistent with the FW mode coupling (Figure 2d).

### 2.3 Nonlinear Wave Equation Model

The effect of the plasmonic MPM in the growth of the THG wave amplitude in its propagation along the waveguide is theoretically modeled by solving the nonlinear Helmholtz equation assuming the validity of the slowly varying amplitude approximation.^[^ ^14^ ^]^ In this case, the second‐order derivative with respect to *z*, the coordinate in the propagation direction, of the THG electric field complex amplitude *A* ^TH^(*z*) is neglected, and the equation is simplified to:

(2) dATHzdz=κAFWz3eizReΔk−ATHzlpTH

where *A* ^FW^(*z*) is the FW field amplitude, *κ* is a proportionality constant, $l_{p}^{\mathrm{TH}}$ is the TH propagation length, and Δ*k* is the phase mismatch between FW and THG. Neglecting the depletion of the FW pump due to the parametric process but keeping linear absorption, the spatial variation of *A* ^FW^(*z*) is simply

(3) $$A^{\mathrm{FW}}\left(z\right)=A_{0}^{\mathrm{FW}}e^{-z/l_{p}^{\mathrm{FW}}}$$

with $l_{p}^{\mathrm{FW}}$ as the FW propagation length and $A_{0}^{\mathrm{FW}}$ as the pump amplitude at *z* = 0 (Antenna‐in, Figure 2a). Solving Equation 2 with boundary condition *A* ^TH^ (*z* = 0) = 0 gives the detected signal, proportional to *S* (*L*, Δ*k*) = |*A* ^TH^(*z* = *L*)|^2^, as:

(4) SL,Δk=lpFWlpTH2e−2LlpTH+e−6LlpFW−2e−L3lpFW+1lpTHcosLReΔklpFW−3lpTH2+lpFWlpTHReΔk2

with *L* as the waveguide length. Within the limit of lossless propagation, Equation 4 reduces to the familiar phase mismatch expression *L* ^2^sinc^2^[Re(Δ*k*) *L*/2].^[^ ^14^ ^]^

**Figure 5:**
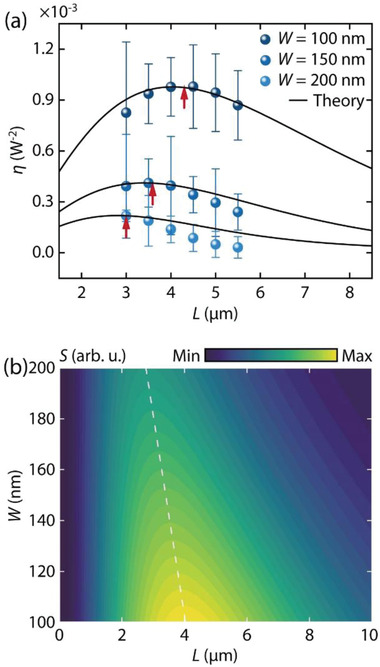
a) THG conversion efficiency (*η*) experimentally evaluated for the waveguides of *W* = 100 nm, 150 nm, and 200 nm, plotted as a function of *L*. Upward arrows represent *L*_eff_ corresponding to the local maxima of *η*. Error bars represent the standard deviation of measurements of five devices considering fabrication deviations. Solid lines show the theoretical evaluation *S*(*L*, Δ*k*) from Equation 4. b) Contour map of *S*(*L*, Δ*k*) from Equation 4, with values of Δ*n* and *l*_p_ from Figure S4a,b (Supporting Information), for 100 < *W* < 200 nm and 0 < *L* < 10 µm. The white dashed line indicates the maxima of *S*(*L*, Δ*k*) values.

To compare the variation of *S*(*L*, Δ*k*) with *L* and Δ*k*, we measured the THG conversion efficiency *η* for devices with *L* ranging from 3 to 5.5 µm, with increments of 0.5 µm, with three representative gap widths *W* = 100, 150, and 200 nm. In general, as *W* is increased from 100 to 200 nm, the phase mismatch Δ*k* between AS_1, FW_ and AS_2, TH_ becomes larger, compromising the spatial overlap of the modes and diminishing the THG efficiency (see Section S4, Supporting Information for the effective index and phase matching plots). **Figure** **5**a shows *η* (spheres) plotted along with the theoretical results (solid lines) obtained by evaluating Equation 4. Individual theoretical curves are rescaled to match the maxima of the experimental data set corresponding to each *W*. Good qualitative agreement is observed between the theory and the experiment. Slight deviations can be attributed to fabrication imperfections. For *W* = 100 nm case, *η* increases with *L*, until a maximum is reached at *L* = *L*_eff_ (upward arrows in Figure 5a), the most well‐suited waveguide length for the coherent accumulation of the THG amplitude to compensate for the propagation losses of the combined TH and FW modes. After this point, the observed signal power starts to decrease as the parametric process ceases to dominate over the losses. For the *W* = 150 nm and 200 nm cases, we also observe similar trends, but the THG conversion peaks are shifted to lower values of *L*_eff_ with reduced power, because of the higher phase mismatch between the TH and FW modes, resulting in faster decoherence. This behavior with varying *W* is obvious in the contour map of *S*(*L*, Δ*k*) plotted in Figure 5b. The values of Δ*n* ( = Δ*k* × *λ* ^TH^/2*π*) reflecting the phase mismatch (in Figure S4a, Supporting Information), obtained from the FDTD mode calculations for 100 < *W* < 200 nm are used for computing the contour plot. The white dashed line highlights the trend in the THG maximum and indicates the variation of the coherent signal accumulation length when MPM is fulfilled with a decrease in *W*. By including the propagation and effective index information obtained from the FDTD simulations, the general trend observed in the THG intensity from the theory shows a consistent agreement with the experiment. We note that by decreasing *W* to below 100 nm the propagation losses start dominating the THG process, which will effectively reduce *L*_eff_ to smaller values, with lower *η*. It is envisioned that other structural parameters such as the antenna coupling factors can be optimized to further enhance THG efficiencies. We note that the efficiency of the Antenna‐out used for the present study is limited to ≈ 0.17% for the THG outcoupling at 517 nm (see Section S4, Supporting Information). Nevertheless, for milliwatt input powers (*P*_in_), we observe *η* ≈ 10^–3^ W^‐2^ over a length scale (*L*_eff_) ≈ 4.3 µm (see Table S1, Supporting Information). Taking into account the antenna efficiencies, it indicates a high internal (within the TWTL waveguide) TH conversion in the plasmonic waveguide before the Antenna‐out, as a result of the MPM achieved here.

## 3 Conclusions 

In summary, we have demonstrated MPM for THG using plasmonic waveguides in the TWTL configuration, with P3HT as the nonlinear medium for the wavelength conversion from 1550 to 517 nm. The gap width between the slabs is chosen to maximize the MPM between the AS modes (AS_1, FW_ and AS_2, TH_) while keeping the incident FW laser beam effectively coupled to the plasmonic modes, resulting in a coherent nonlinear interaction in the waveguide to accumulate the THG signal. As the MPM is strongly correlated with the symmetry of the modes, THG can be directly modulated with the incident laser polarization angle *θ*_in_. We observe maxima in the THG signal for *θ*_in_= 90^○^ and 270^○^ when the AS modes are efficiently excited in the waveguide. The emitted power follows the input power in the third order, consistent with the typical TH response. Numerical calculations performed using nonlinear FDTD and wave‐equation studies corroborate the experimental observations. The case of *W* = 100 nm with *A*_eff_ ≈ 0.11 µm^2^ and *L*_eff_ ≈ 4.3 µm ensuing a local maximum in *η* ≈ 10^–3^ W^‐2^ (this efficiency is similar to that of other devices based on NOPs^[^ ^43^, ^44^ ^]^) demonstrates that the tradeoff between the THG and the plasmonic propagation losses within µm scale interaction length can be mitigated. In other words, based on our analysis we anticipate that the device performance can be further improved for even smaller devices provided Δ*k* is further minimized (see Figure 2b). In addition, the overall efficiency of the devices can be improved by optimizing the impedance matching between guided THG modes and radiation at the Antenna‐out. Our present study provides a solution for nonlinear optical conversion based on compact plasmonic waveguides that enable the tunability of phase‐matching via geometry and polarization control.

## 4 Experimental and Simulation Section

### Fabrication

Plasmonic waveguides were fabricated on a 285 nm SiO_2_–Si substrate, using electron beam lithography (EBL) and e‐beam evaporation. The waveguides were defined by EBL (JEOL, JBX‐6300FS), with bilayer PMMA resist (PMMA 495 A3 and PMMA 950 A5, prebaked at 180 °C for 5 min and 2 min respectively), followed by resist development in MIBK:IPA (1:3) for 30 s and IPA for 30 s. After development, the Ag layer (80 nm thick) with a Ti adhesion layer (3 nm thick) was deposited using e‐beam evaporation (AJA, Orion 8E) followed by lift‐off in acetone. The P3HT layer was first created by dissolving 20 mg of regioregular P3HT (average molecular weight MW = 70000–100000 g mole^‐1^, Sigma‐Aldrich) in 1 mL of chlorobenzene. The solution was then spin‐casted on the samples at 500 rpm for 10 s, then at 1000 rpm for 60 s to create a 200 nm film. The devices were thermally annealed in air at 100 °C for 10 min to stabilize the optical performance.

### Numerical Simulations

The eigenmode profiles for the plasmonic waveguides were simulated using the FDTD method (Lumerical^[^ ^41^ ^]^ FDTD Solutions, Ansys Canada Ltd.). The optical properties of Ag (80 nm), SiO_2_ (285 nm), and Si were taken from Palik.^[^ ^45^ ^]^ The optical properties of the P3HT cladding (200 nm) were obtained from the ellipsometry measurements as detailed in Section S5 (Supporting Information). For the effective index calculation, a 2D cross‐section of the waveguide in the *xy*‐plane was used (see Figure 1 inset), with a simulation domain size of 1.5 µm × 0.75 µm (*x* × *y*). A minimum mesh size of d*x* = d*y* = 2 nm was applied across the simulation area. To solve for the AS modes, antisymmetric boundary conditions were used in the ±*x* direction, whereas perfectly matched layers were used as the ±*y* boundary conditions with 12 *Standard* absorbing layers. The THG response of the device was calculated using the nonlinear FDTD method for a 3D domain (domain size 7 µm × 5 µm × 1.5 µm (*x* × *y* × *z*)). Nonlinear material implementation^[^ ^46^ ^]^ of the Lumerical was used to represent the P3HT material. For THG, the polarization *P* in the small field amplitude limit can be written as $P=\varepsilon_{0}E_{x}\left(\omega \right)\left[\chi_{\mathrm{FW}}^{\left(1\right)}+\chi_{\mathrm{TH}}^{\left(3\right)}{\left[E_{x}\left(\omega \right)\right]}^{2}+\cdot \cdot \cdot \right]$. For the convergence of the FDTD model, nonlinear terms have to be small. In other words, $\chi_{\mathrm{TH}}^{\left(3\right)}{\left[E_{x}\left(\omega \right)\right]}^{2}$ should be much smaller than $\chi_{\mathrm{FW}}^{\left(1\right)}$. A $\chi_{\mathrm{TH}}^{\left(3\right)}$ value of ≈ 10^–19^ m^2^ V^‐2^ was used for the P3HT film and a field peak amplitude of ≈ 10^7^ V m^‐1^ for the incident Gaussian beam, which gives $\chi_{\mathrm{TH}}^{\left(3\right)}{\left|E\right|}^{2}$ ≈ 10^–5^. This value is much smaller than $\chi_{\mathrm{FW}}^{\left(1\right)}$ (typically ≈ 1) and guarantees the convergence of the numerical simulations. Perfectly matched layers were used as the boundary conditions in all three directions and non‐uniform meshing was applied across the simulation domain for the nonlinear FDTD simulations.

### Optical Measurements

The laser beam at 1550 nm (FW) with an fwhm spot size of ≈ 1.2 µm and average power of ≈ 10 mW produced from a femtosecond fiber laser system (Toptica, FemtoFiber pro, 1550 nm, 90 fs, 80 MHz) was focused on the Antenna‐in (Figure 1) through an objective (Leica, 100×, NA 0.85). To spatially monitor the THG along the waveguide (i.e., *z*‐axis) one used a Y‐fiber (Thorlabs, TW560R1F1) mounted on a 3D piezo stage (Piezojena Tritor 100) tandem on a 3D manual mechanical stage. The input port of the fiber was mounted on the microscope to collect the signal focused by a lens (Lens A, Nikon 20×, NA 0.5). The signal port (99%) was connected to the spectrometer, while the tap port (1%) was coupled to a 532 nm laser. The location and area (with a detection spot diameter of ≈ 0.7 µm) of the signal being collected and measured are known by observing the 532 nm laser spot on the sample with the imaging branch of the microscope. By moving the input port of the fiber controllably with the 3D piezo stage, the line‐trace THG spectra were recorded. The spectrometer (Andor, Kymera 328i) equipped with a Vis‐detector (Andor, iDus 401A) was used to measure the spectra. With the visible sCMOS camera (PCO, Edge 4.2) on the other branch, the distribution of light emission intensity was captured. The collection efficiency of the setup to the sCMOS (QE = 0.75, gain = 0.46 e‐ per count^[^ ^47^ ^]^) for 517 nm is estimated to be 16.9%, hence the detection sensitivity (*S*_d_) is 1.4×10^–3^ fW s count^–1^. THG power (*P*_out_) is converted from the time‐averaged intensity (*I*_TH_) collected by the sCMOS through *P*_out_ = *S*_d_ × *I*_TH_.

## Conflict of Interest

The authors declare no conflict of interest.

## Supporting information

Supporting Information

## Data Availability

The data that support the findings of this study are available from the corresponding author upon reasonable request.

## References

[1] D. Cotter , R. J. Manning , K. J. Blow , A. D. Ellis , A. E. Kelly , D. Nesset , I. D. Phillips , A. J. Poustie , D. C. Rogers , Science 1999, 286, 1523. 10567251 10.1126/science.286.5444.1523

[2] B. Vanus , C. Baker , L. Chen , X. Bao , Opt. Express 2020, 28, 3789. 32122040 10.1364/OE.384004

[3] M. Mesch , B. Metzger , M. Hentschel , H. Giessen , Nano Lett. 2016, 16, 3155. 27050296 10.1021/acs.nanolett.6b00478

[4] W. R. Zipfel , R. M. Williams , W. W. Webb , Nat. Biotechnol. 2003, 21, 1369. 14595365 10.1038/nbt899

[5] F. Pincella , K. Isozaki , K. Miki , Light: Sci. Appl. 2014, 3, e133.

[6] M. Marchini , A. Gualandi , L. Mengozzi , P. Franchi , M. Lucarini , P. G. Cozzi , V. Balzani , P. Ceroni , Phys. Chem. Chem. Phys. 2018, 20, 8071. 29516066 10.1039/c7cp08011e

[7] L. Caspani , C. Xiong , B. J. Eggleton , D. Bajoni , M. Liscidini , M. Galli , R. Morandotti , D. J. Moss , Light: Sci. Appl. 2017, 6, e17100. 30167217 10.1038/lsa.2017.100 PMC6062040

[8] M. Geissbuehler , L. Bonacina , V. Shcheslavskiy , N. L. Bocchio , S. Geissbuehler , M. Leutenegger , I. Märki , J.‐P. Wolf , T. Lasser , Nano Lett. 2012, 12, 1668. 22372559 10.1021/nl300070n

[9] S. Witte , A. Negrean , J. C. Lodder , C. P. J. de Kock , G. Testa Silva , H. D. Mansvelder , M. Louise Groot , Proc. Natl. Acad. Sci. USA 2011, 108, 5970. 21444784 10.1073/pnas.1018743108 PMC3076839

[10] D. Débarre , W. Supatto , A.‐M. Pena , A. Fabre , T. Tordjmann , L. Combettes , M.‐C. Schanne‐Klein , E. Beaurepaire , Nat. Methods 2006, 3, 47. 16369553 10.1038/nmeth813

[11] J. Morizet , G. Ducourthial , W. Supatto , A. Boutillon , R. Legouis , M.‐C. Schanne‐Klein , C. Stringari , E. Beaurepaire , Optica 2019, 6, 385.

[12] I. Alonso Calafell , L. A. Rozema , D. Alcaraz Iranzo , A. Trenti , P. K. Jenke , J. D. Cox , A. Kumar , H. Bieliaiev , S. Nanot , C. Peng , D. K. Efetov , J.‐Y. Hong , J. Kong , D. R. Englund , F. J. García de Abajo , F. H. L. Koppens , P. Walther , Nat. Nanotechnol. 2021, 16, 318. 33318642 10.1038/s41565-020-00808-w

[13] K. I. Okhlopkov , P. A. Shafirin , A. A. Ezhov , N. A. Orlikovsky , M. R. Shcherbakov , A. A. Fedyanin , ACS Photonics 2019, 6, 189.

[14] R. W. Boyd , Nonlinear Optics, Academic, San Diego, CA 2008.

[15] L. Chang , A. Boes , X. Guo , D. T. Spencer , M. J. Kennedy , J. D. Peters , N. Volet , J. Chiles , A. Kowligy , N. Nader , D. D. Hickstein , E. J. Stanton , S. A. Diddams , S. B. Papp , J. E. Bowers , Laser Photonics Rev. 2018, 12, 1800149.

[16] R. Luo , Y. He , H. Liang , M. Li , Q. Lin , Laser Photonics Rev. 2019, 13, 1800288.

[17] J. A. Schuller , E. S. Barnard , W. S. Cai , Y. C. Jun , J. S. White , M. L. Brongersma , Nat. Mater. 2010, 9, 193. 20168343 10.1038/nmat2630

[18] T. Huang , X. Shao , Z. Wu , T. Lee , T. Wu , Y. Sun , J. Zhang , H. Q. Lam , G. Brambilla , P. P. Shum , IEEE Photonics J 2014, 10.1109/jphot.2014.2323302.

[19] M. Kauranen , A. V. Zayats , Nat. Photonics 2012, 6, 737.

[20] R. F. Oulton , V. J. Sorger , T. Zentgraf , R. M. Ma , C. Gladden , L. Dai , G. Bartal , X. Zhang , Nature 2009, 461, 629. 19718019 10.1038/nature08364

[21] C. Reimer , M. Kues , P. Roztocki , B. Wetzel , F. Grazioso , B. E. Little , S. T. Chu , T. Johnston , Y. Bromberg , L. Caspani , D. J. Moss , R. Morandotti , Science 2016, 351, 1176. 26965623 10.1126/science.aad8532

[22] R. W. Boyd , Z. Shi , I. De Leon , Opt. Commun. 2014, 326, 74.

[23] A. V. Zayats , I. I. Smolyaninov , A. A. Maradudin , Phys. Rep. 2005, 408, 131.

[24] X. Lin , J. Ye , Y. Yan , H. Dong , J. Gu , W. Zhang , C. Wei , J. Yao , Y. S. Zhao , Mater. Chem. Front. 2018, 2, 491.

[25] Z. Li , B. Corbett , A. Gocalinska , E. Pelucchi , W. Chen , K. M. Ryan , P. Khan , C. Silien , H. Xu , N. Liu , Light: Sci. Appl. 2020, 9, 180. 33110598 10.1038/s41377-020-00414-4 PMC7582155

[26] J. Shi , Y. Li , M. Kang , X. He , N. J. Halas , P. Nordlander , S. Zhang , H. Xu , Nano Lett. 2019, 19, 3838. 31125243 10.1021/acs.nanolett.9b01004

[27] J. Shi , X. He , W. Chen , Y. Li , M. Kang , Y. Cai , H. Xu , Nano Lett. 2022, 22, 688. 35025516 10.1021/acs.nanolett.1c03824

[28] T. W. Baehr‐Jones , M. J. Hochberg , J. Phys. Chem. C 2008, 112, 8085.

[29] J. Leuthold , W. Freude , J. M. Brosi , R. Baets , P. Dumon , I. Biaggio , M. L. Scimeca , F. Diederich , B. Frank , C. Koos , Proc. IEEE 2009, 97, 1304. 10.1364/OE.17.017357 19907521

[30] C. Koos , P. Vorreau , T. Vallaitis , P. Dumon , W. Bogaerts , R. Baets , B. Esembeson , I. Biaggio , T. Michinobu , F. Diederich , W. Freude , J. Leuthold , Nat. Photonics 2009, 3, 216.

[31] W. Cai , A. P. Vasudev , M. L. Brongersma , Science 2011, 333, 1720. 21940887 10.1126/science.1207858

[32] A. Melikyan , L. Alloatti , A. Muslija , D. Hillerkuss , P. C. Schindler , J. Li , R. Palmer , D. Korn , S. Muehlbrandt , D. Van Thourhout , B. Chen , R. Dinu , M. Sommer , C. Koos , M. Kohl , W. Freude , J. Leuthold , Nat. Photonics 2014, 8, 229.

[33] J. M. Hales , S. Barlow , H. Kim , S. Mukhopadhyay , J.‐L. Brédas , J. W. Perry , S. R. Marder , Chem. Mater. 2014, 26, 549.

[34] Y. Wang , S. He , X. Gao , P. Ye , L. Lei , W. Dong , X. Zhang , P. Xu , Photonics Res. 2022, 10, 50.

[35] M. P. Nielsen , X. Shi , P. Dichtl , S. A. Maier , R. F. Oulton , Science 2017, 358, 1179. 29191907 10.1126/science.aao1467

[36] M. Ochs , L. Zurak , E. Krauss , J. Meier , M. Emmerling , R. Kullock , B. Hecht , Nano Lett. 2021, 21, 4225. 33929199 10.1021/acs.nanolett.1c00182

[37] P. Geisler , G. Razinskas , E. Krauss , X.‐F. Wu , C. Rewitz , P. Tuchscherer , S. Goetz , C.‐B. Huang , T. Brixner , B. Hecht , Phys. Rev. Lett. 2013, 111, 183901. 24237520 10.1103/PhysRevLett.111.183901

[38] D. B. Anderson , J. T. Boyd , Appl. Phys. Lett. 1971, 19, 266.

[39] H. Kishida , K. Hirota , T. Wakabayashi , H. Okamoto , H. Kokubo , T. Yamamoto , Appl. Phys. Lett. 2005, 87, 121902.

[40] K. M. McPeak , S. V. Jayanti , S. J. P. Kress , S. Meyer , S. Iotti , A. Rossinelli , D. J. Norris , ACS Photonics 2015, 2, 326. 25950012 10.1021/ph5004237 PMC4416469

[41] Lumerical Inc, https://support.lumerical.com/hc/en-us/articles/1500007184901-Lumerical-Citation-Instruction.

[42] C. Koos , L. Jacome , C. Poulton , J. Leuthold , W. Freude , Opt. Express 2007, 15, 5976. 19546900 10.1364/oe.15.005976

[43] G. Ramos‐Ortiz , M. Cha , S. Thayumanavan , J. C. Mendez , S. R. Marder , B. Kippelen , Appl. Phys. Lett. 2004, 85, 179.

[44] J. Kim , C. H. S. S. P. Kumar , M. Cha , H. Choi , K.‐J. Kim , N. Peyghambarian , Sci. Rep. 2018, 8, 16419. 30401913 10.1038/s41598-018-34788-8 PMC6219598

[45] E. D. Palik , Handbook of Optical Constants of Solids, Academic Press, Burlington 1997.

[46] A. Taflove , S. C. Hagness , Computational Electrodynamics: The Finite‐Difference Time‐Domain Method, Artech House, Boston 2005.

[47] https://www.pco.de/fileadmin/user_upload/pco‐product_sheets/DS_PCOEDGE42_V202.pdf (accessed: August 2021).

