# Supplementary material for: Phase Matching via Plasmonic Modal Dispersion for Third Harmonic Generation
Source: Adv Sci (Weinh). 2022 Jun 5;9(21):2201180. doi: 10.1002/advs.202201180 (PMC9313513; doi:10.1002/advs.202201180)
Supplement: Supplementary file 1 — Supporting Information [file ADVS-9-2201180-s001.pdf]

ADVANCED SCIENCE
Open Access

Supporting Information

for *Adv. Sci.*, DOI: 10.1002/advs.202201180

Phase Matching via Plasmonic Modal Dispersion for Third Harmonic Generation

Zhe Wang, Zhe Wang, Vijith Kalathingal, Yi Wei Ho, Thanh Xuan Hoang, Hong-Son Chu, Yongxin Guo, José C. Viana-Gomes, Goki Eda, and Christian A. Nijhuis**

SUPPORTING INFORMATION

Phase Matching via Plasmonic Modal Dispersion for Third Harmonic Generation

Zhe Wang[†,1], *Zhe Wang*[†,2], *Vijith Kalathingal*[†,3], *Yi Wei Ho*[†,2], *Thanh Xuan Hoang*[4], *Hong-Son Chu*[4], *Yongxin Guo*[3], *José C. Viana-Gomes**[,2,5,6], *Goki Eda*[1,2,6], *and Christian A. Nijhuis**[,1,6,7]

[1]Department of Chemistry, National University of Singapore, 3 Science Drive 3, Singapore 117543, Singapore

[2]Department of Physics, National University of Singapore, 2 Science Drive 3, Singapore 117542, Singapore

[3]Department of Electrical and Computer Engineering, National University of Singapore, 4 Engineering Drive 3, 117583, Singapore

[4]Department of Electronics and Photonics, Institute of High Performance Computing, A*STAR (Agency for Science, Technology and Research), 1 Fusionopolis Way, #16-16 Connexis, Singapore 138632, Singapore

[5]Department of Physics of University of Minho, and Physics Center of Minho and Porto Universities (CF-UM-UP), Campus of Gualtar, 4710-057 Braga, Portugal

[6]Centre for Advanced 2D Materials and Graphene Research Centre, National University of Singapore, 6 Science Drive 2, Singapore 117546, Singapore

[7]Hybrid Materials for Opto-Electronics Group, Department of Molecules and Materials, MESA+ Institute for Nanotechnology and Center for Brain-Inspired Nano Systems, Faculty of Science and Technology, University of Twente, 7500 AE Enschede, The Netherlands

[†] These authors contributed equally.

* Authors to whom correspondence should be addressed: c.a.nijhuis@utwente.nl, zgomes@fisica.uminho.pt

S1. Atomic force microscope images

Figure S1 shows the atomic force microscope (AFM, Bruker FastScan) images for the waveguide and the spin-cast P3HT film on the SiO_2-Si substrate, and the height profiles are plotted in the bottom panels, respectively. To measure the thickness of the P3HT film, we scratched the film using a plastic tweezer to form a step edge. The step height was profiled across the scratch using AFM, as is shown in Figure S1b. The estimated film thickness is ~200 nm.

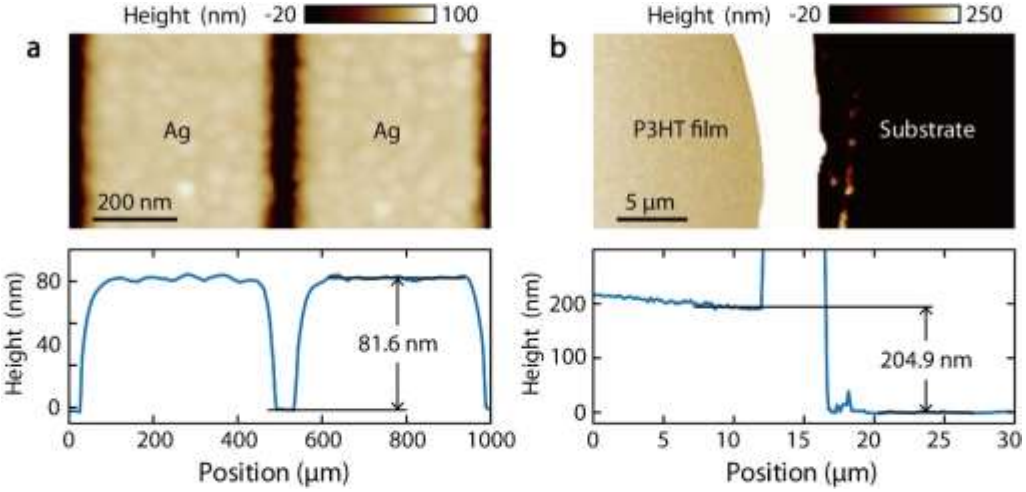

Figure S1. AFM images of (**a**) the waveguide and (**b**) the spin-cast P3HT film on the SiO_2-Si substrate. The film thickness was measured across the scratch.

S2. Material dispersion of P3HT film

The refractive index of the as-deposited P3HT film on the SiO_2-Si substrate was measured using an ellipsometer (Accurion, Nanofilm EP4[1]) equipped with a Vis-detector. The ellipsometric measurement was performed over a wavelength range of 400 – 900 nm, in a reflection mode with multiple angles of incidence ranging from 40° to 70° to the surface normal, in steps of 5°. The film was formed in good uniformity, without biaxial anisotropy within the

plane of the film confirmed by rotating the sample in the plane of the film. Data analysis over the measured phase difference (Δ) and amplitude (Ψ) signals was performed in the EP4Model software (Accurion). The P3HT film was modeled as a homogeneous layer with a complex refractive index *n* and a thickness *h*. The estimated film thickness was ~ 200 nm, which agrees with the AFM measurement shown in Figure S1b. The real and imaginary parts of the refractive index obtained from the ellipsometry analysis for the P3HT film are shown in Figure S2. By extrapolating the Re(*n*) curve in Figure S2 to the wavelength range around 1550 nm, the average refractive index of the P3HT film was estimated to be ~1.7, with low absorption losses.

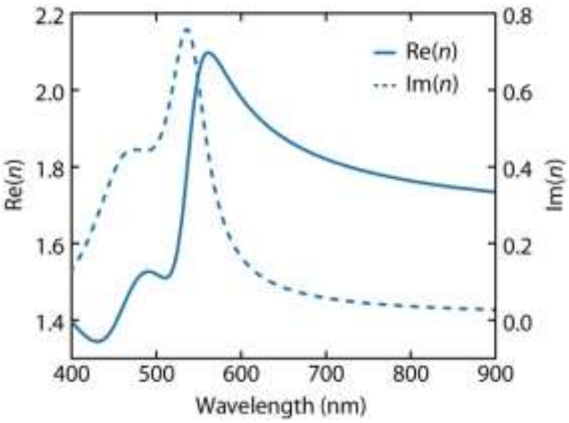

Figure S2. The wavelength-dependent refractive index of the P3HT film.

S3. Charge distribution of the modes

The eigenmodes supported by the TWTL waveguides were designated with specific orders according to the charge distribution (ρ) in the Ag slabs. Figure S3 illustrates ρ of the $AS_{1,\,FW}$ (top) and $AS_{2,\,TH}$ (bottom) modes, overlaid with the electric field vector distribution (arrows), depicting the antisymmetry in ρ with respect to the y-axis.

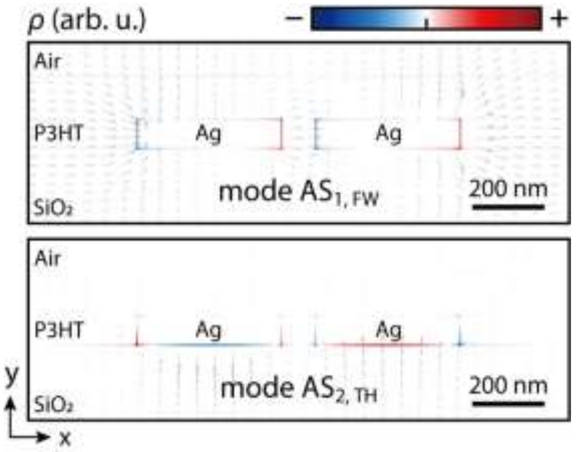

Figure S3. Charge distribution (ρ) of the $AS_{1,\,FW}$ (for 1550 nm) and $AS_{2,\,TH}$ (for 517 nm) modes overlaid with electric field vector distribution (arrows).

S4. Phase mismatch and propagation lengths

The effective refractive index obtained from the simulations for the $AS_{1,\,FW}$ and $AS_{2,\,TH}$ modes as a function of gap width is plotted in Figure S4a (left *y*-axis). Phase mismatch ($n_{\mathrm{eff}}^{\mathrm{TH}} - n_{\mathrm{eff}}^{\mathrm{FW}}$) estimated from the effective index values are shown in the right *y*-axis. Figure S4b shows the propagation lengths l_{p} and the propagation losses of the two investigated modes. The propagation length is defined as $l_{\mathrm{p}}^{\alpha} = 1/(2\mathrm{Im}[k^{\alpha}])$, over which the SPP power loses 1/e. Similarly, the propagation loss can be given by $loss^{\alpha} = -20\log_{10}\left(e^{-\mathrm{Im}[k^{\alpha}]}\right)$. Figure S4c shows the simulated coupling efficiencies for the input (1550 nm) and the output (517 nm) antennas. Figure S4d shows the simulated far-field radiation patterns for the $AS_{1,\,FW}$ and $AS_{2,\,TH}$ modes from the Antenna-out location, indicating a nearly vertical ($-10° < \theta_{\mathrm{out}} < +10°$) outcoupling for the $AS_{2,\,TH}$ mode with respect to the waveguide orientation (Figure S4d inset), which can be captured by an objective lens of NA = 0.85.

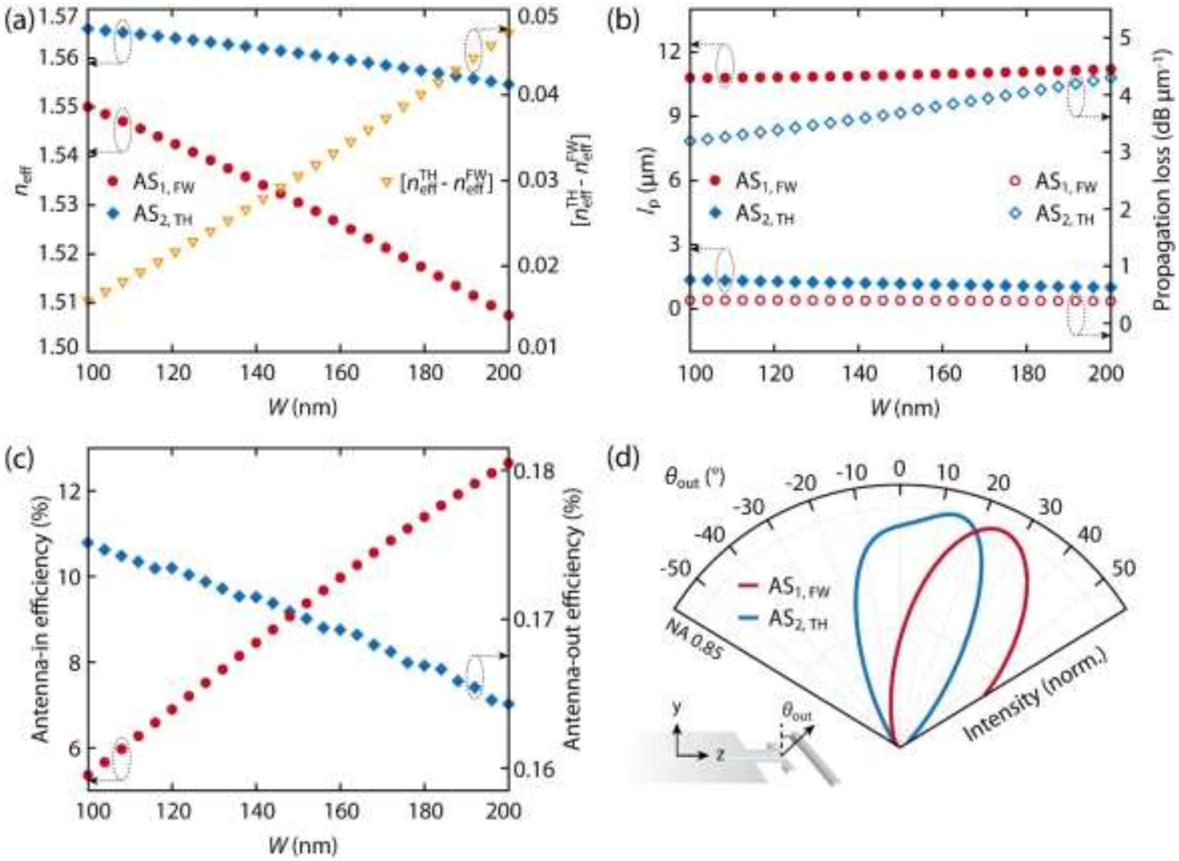

Figure S4. (**a**) Effective refractive index n_{eff} of plasmonic modes $AS_{1,\ FW}$ and $AS_{2,\ TH}$, and the index mismatch $n_{\text{eff}}^{\text{TH}} - n_{\text{eff}}^{\text{FW}}$ between them as a function of gap width *W*. (**b**) The propagation lengths l_{p} (left-y axis, solid red circle for mode $AS_{1,\ FW}$, solid blue diamond for mode $AS_{2,\ TH}$) and the propagation losses (right-y axis, hollow red circle for mode $AS_{1,\ FW}$, hollow blue diamond for mode $AS_{2,\ TH}$) of the two investigated modes. (**c**) Coupling efficiencies for Antenna-in and Antenna-out. (**d**) Far-field radiation intensity from the Antenna-out for the $AS_{1,\ FW}$ (red solid curve) and $AS_{2,\ TH}$ (blue solid curve) modes, plotted as a function of the detection angle (θ_{out}) in the *yz*-plane (inset). NA= 0.85 demarcates the maximum collection angle ($\pm$58.2^{o}) of the objective.

S5. Nonlinear optical susceptibility of P3HT film

The third-order nonlinear optical susceptibility coefficient $\chi^{(3)}_{\mathrm{P3HT}}$ of the P3HT film was calculated using the model deduced in ref. [2]. The THG in reflection with the FW beam pumping the sample:

$$P_{\mathrm{TH}}(3\omega) = \frac{64\sqrt{3}S^2\left|\chi^{(3)}_{\mathrm{P3HT}}\right|^2\omega^2}{c^2\varepsilon_0^2(RR\,\Delta\tau\,\pi\,r^2)^2(1+n_{\mathrm{s}})^8}P^3(\omega) \tag{S1}$$

Where $P_{\mathrm{TH}}(3\omega)$ and $P(\omega)$ are the average powers for THG and pump, respectively. S = 0.94 is a shape factor for Gaussian pulses, ω is the frequency of the pump beam, c is the speed of light in vacuum, RR is the repetition rate (80 MHz), r is the spot radius, n_{s} is the refractive index of the substrate. Therefore $\chi^{(3)}_{\mathrm{P3HT}}$ is expressed as:

$$\chi^{(3)}_{\mathrm{P3HT}} = \sqrt{\frac{P_{\mathrm{THG}}(3\omega)}{P^3(\omega)}\frac{c^2\varepsilon_0^2(RR\,\Delta\tau\,\pi\,r^2)^2(1+n_{\mathrm{s}})^8}{64\sqrt{3}S^2\omega^2}} \tag{S2}$$

Since the experiment is under the same condition, which means:

$$\frac{\chi^{(3)}_{\mathrm{P3HT}}}{\chi^{(3)}_0} = \sqrt{\frac{P_{\mathrm{P3HT}}(3\omega)}{P_0(3\omega)}} \tag{S3}$$

where $\chi^{(3)}_0$ can be the third-order nonlinear optical susceptibility coefficient of a known material. Here we apply the WS_2 monolayer in the calculation whose nonlinear coefficient $\chi^{(3)}_{\mathrm{WS_2}}$=2.4×10^{-19} m^2V^{-2} has been well-characterized and reported.[3] Figure S5 shows the measured THG signals from the P3HT film and a known material WS_2 monolayer. From which we finally extract the nonlinear parameter of $\chi^{(3)}_{\mathrm{P3HT}}$= 0.71 × 10^{-19} m^2 V^{-2} (= 5.08 × 10^{-12} esu), which is comparable to typically reported values. We note that the conjugate polythiophene structures favor THG more than SHG ($\chi^{(2)}$ ~ 10^{-30} esu).[4] As the structure does not follow the typical donor-conjugate-acceptor (D-π-A) paradigm of second-order nonlinear optical polymers, it lacks a dipole-allowed

bulk $\chi^{(2)}$ susceptibility. The $\chi^{(2)}$ susceptibility of P3HT films has to come from surface effects or has to be related to the magnetic dipole or electric quadrupole terms.

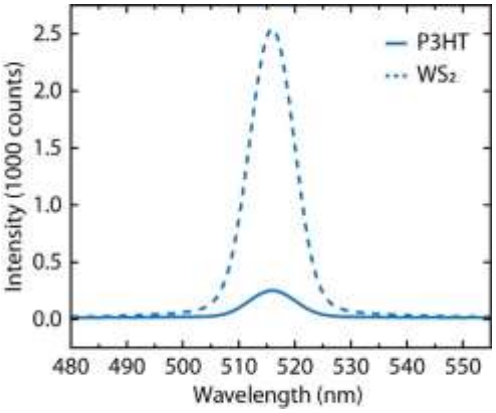

Figure S5. THG spectra of the off-device P3HT film and WS_2 monolayer excited at 1550 nm.

S6. THG efficiency

Table S1 lists A_{eff} (Equation 1), L_{eff} (upward arrows in Figure 5a), P_{in}, P_{out} and η $(= P_{\mathrm{out}}/(P_{\mathrm{in}})^3)$ for waveguides with W = 100, 150, and 200 nm. Note that the average input power (~ 1 mW (10^5 W cm^{-2})) is four orders of magnitude lower than the irreversible bleaching threshold for P3HT (~ 10^9 W cm^{-2}).[5]

Table S1. THG efficiencies for waveguides with W = 100, 150, and 200 nm

W (nm)	A_{eff} (μm^2)	L_{eff} (μm)	P_{in} (mW)	P_{out} (fW)	η (10^{-3} W^{-2})
100	0.11	4.3	0.6	211	0.98
150	0.12	3.6	0.6	89	0.41
200	0.14	3.0	0.6	47	0.22

References

[1] https://accurion.com/thin-film-characterization/products/nanofilm-ep4.

[2] R. Woodward, R. Murray, C. Phelan, R. De Oliveira, T. Runcorn, E. Kelleher, S. Li, E. De Oliveira, G. Fechine, G. Eda, *2D Materials* **2016**, *4*, 011006.

[3] A. Autere, H. Jussila, Y. Dai, Y. Wang, H. Lipsanen, Z. Sun, *Adv. Mater.* **2018**, *30*, 1705963.

[4] S. Deckers, S. Vandendriessche, D. Cornelis, F. Monnaie, G. Koeckelberghs, I. Asselberghs, T. Verbiest, M. Van Der Veen, *Chem. Commun.* **2014**, *50*, 2741.

[5] J. Szeremeta, R. Kolkowski, M. Nyk, M. Samoc, *J. Phys. Chem. C* **2013**, *117*, 26197.
